# A Fermented Wheat Germ Extract Contains Protein Components Active against NSCLC Xenografts In Vivo

**DOI:** 10.3390/cimb45090448

**Published:** 2023-08-25

**Authors:** Daniel J. Levis, Joshua F. Meckler, Robert T. O’Donnell, Joseph M. Tuscano

**Affiliations:** 1 Division of Hematology and Oncology, Department of Internal Medicine, University of California Davis School of Medicine, Sacramento, CA 95817, USA; djlevis@ucdavis.edu (D.J.L.);; 2 Department of Veterans Affairs, Northern California Healthcare System, Sacramento, CA 95652, USA

**Keywords:** non-small cell lung cancer (NSCLC), fermented wheat germ protein (FWGP), fermented wheat germ extract, A549, non-toxic, natural product

## Abstract

Non-small cell lung cancer (NSCLC) continues to be the leading cause of cancer-related deaths. Although advances have been made in the past decade to treat such tumors, most options induce multiple side effects, and many patients discontinue therapy due to toxicity. Thus, the need remains for non-toxic, effective NSCLC therapies, especially in an elderly patient population. Our lab has previously identified a protein fraction from the nutraceutical Avemar^®^—dubbed fermented wheat germ protein (FWGP)—with demonstrated efficacy in lymphoma models both in vitro and in vivo. Here, we show that FWGP also has anti-tumor activity in vitro and in vivo against lung cancer. In vitro cytotoxicity against multiple lung cancer cell lines yielded IC_50_ values comparable to those previously established with the parent product, Avemar. Further, significant A549 xenograft growth inhibition occurred in athymic nu/nu mice receiving FWGP in both pre-radiated and non-radiated models when compared to the untreated control. Encouragingly, mice treated with FWGP experienced no toxicities as detected by weight reduction or blood chemistry analysis. These data support the further study of FWGP as a potential non-toxic therapy for lung cancer and other oncologic indications.

## 1. Introduction

Lung cancer is the leading cause of cancer-related death in men, and the second, following breast cancer, in women [1]. About 75% of patients are diagnosed with lung cancer at advanced stage disease after potentially curative (surgical) intervention is no longer feasible [1]. The 5-year survival rate of 61% for localized (early stage) non-small cell lung carcinoma (NSCLC) patients drops dramatically to about 7% for those whose cancers have metastasized to distant organs [1]. Furthermore, it is common for metastatic disease involving remote locations (i.e., liver, brain, bone) to be detected long before identifying the primary lung lesion [2]. NSCLC accounts for 85% of lung cancers and is associated with “rapid fatal spread”.

Current therapeutic approaches for lung cancer include surgery, radiation therapy, chemotherapy, and immunotherapy such as checkpoint inhibitors. Although they can be effective, checkpoint inhibitors targeting PD-1/L1 are only effective in a subset of patients, often leading to the use of chemotherapeutic combinations. Indeed, NSCLC patients with a PD-L1 tumor score >50% (most likely to benefit) receiving pembrolizumab monotherapy in the landmark phase 3 Keynote-042 clinical trial only experienced a 39% objective response rate (ORR) in a first-line setting. Those with a lower PD-L1 score fared even worse (27% ORR) [3]. Typical side effects of these standard treatments for NSCLC include diarrhea, peripheral neuropathy, hair loss, increased susceptibility to infection, mouth sores, dry skin, liver changes, and reduced quality of life. These effects are especially burdensome to the typically older patient population, who may decide against therapy because of its toxicity. Therefore, effective, less toxic treatment approaches are needed, especially for the elderly.

Fermented wheat germ extract (FWGE), or Avemar, is an over-the-counter nutritional supplement that has been utilized by cancer patients since the discovery of its anticancer properties [4]. There is a wealth of published data demonstrating the ability of FWGE to inhibit cancer cell growth in vitro in malignancies such as ovarian, hepatocellular, T cell leukemia and B cell lymphoma, melanoma, squamous cell oral cancer, breast cancer and colon cancer as a monotherapy or when combined with other agents [5,6,7,8,9,10,11,12]. Control of tumor growth has been demonstrated in animal models of melanoma, colon cancer, and lymphoma [4,10,12,13]. Additionally, in an early clinical trial in melanoma patients, an FWGE + alkylating agent dacarbazine combination demonstrated substantial clinical benefits in terms of progression-free survival and overall survival when compared to dacarbazine alone in the adjuvant setting [14]. Our lab has previously described an active protein formulation derived from FWGE, which we have named fermented wheat germ proteins (FWGP). FWGP has demonstrated potent in vivo efficacy against non-Hodgkin lymphoma (NHL) without appreciable toxicity, and it preferentially kills tumor vs. non-tumor B cells in vitro [12]. Early studies suggest that FWGE has multiple anti-cancer and immunological effects such as cell cycle arrest, regulation of the MHC 1 complex, apoptosis, and induced oxidative stress within malignant cells [11,13,15,16,17]. Malignant cells treated with FWGE have been shown to have reduced glucose consumption and induced pentose phosphate pathways through the inhibition of enzymes such as lactate dehydrogenase, transketolase, and glucose 6-phosphate dehydrogenase [18,19,20]. More recent work by Bencze et al. using a methanol extract of FWGE similarly support the metabolic modulation away from glycolysis—which is increased in cancer cells—and towards oxidative phosphorylation, which is associated with increased release of cytochrome c during apoptosis [21]. We suspect that FWGP, as a derivative of FWGE, may possess many of the same anti-cancer mechanisms.

FWGE is likely comprised of hundreds if not thousands of compounds [9]. One other group has published mass spectroscopy data from FWGE, yielding active proteins such as serpin, peroxidases, and glutenins, among others [22]. Our group, working with a protein formulation of FWGE, have begun to identify components within crude FWGP using mass spectroscopy and have found active fractions after applying FWGP to a Sephadex column. Indeed, our initial mass spectroscopy data of these active fractions has yielded evidence of over 300 proteins including similar proteins as Zhang et al. but also including additional oxidases (i.e., super oxide dismutase), and reductases (i.e., glutathione reductase), among others (mass spectroscopy data not shown).

Fermenting raw wheat germ with *Saccharomyces cerevisiae* releases stable quinone and ascorbic radicals which, along with fibers, phytic acids and proteins, likely contribute to the broad activity towards cancer cells and immune cells. Early evidence suggests the rate of decay or quenching of these stable radicals is proportional to the survival of Ehrlich ascites tumor cells, suggesting a direct cytotoxic mechanism [23]. Zhang et al., quantified total sugars, phenols, lactic acid, protein and 2,6-dimethoxy benzoquinone (DMBQ) after fermentation with a lactic acid bacterium (*Lactobacilllus plantarum*), noting the increase in phenol, lactic acid, protein and a 3-fold increase in DMBQ content [22]. Although there is evidence that DMBQ is an active component in crude FWGE, we and others have reported on the activity of protein components (FWGP), suggesting there may be other active molecules in FWGE outside of DMBQ. FWGE treated with proteinase K or heat showed markedly lower cytotoxicity toward cancer cells in vitro when compared to untreated FWGE [12]. Additionally, fractions of FWGP applied to size exclusion columns where small molecules such as DMBQ are removed still showed significant cytotoxicity in in vitro and in vivo lymphoma models. Our group has previously shown that the efficacy of FWGP is mediated in part, by the activation of natural killer cells and induction of tumor cell apoptosis [12]. In this study, we sought to demonstrate that FWGP, in addition to being effective against NHL, has in vitro and in vivo growth inhibition of NSCLC. Future studies will include more in-depth research into additional anti-cancer mechanisms of FWGP in various lung cancer models.

## 2. Results

### 2.1. FWGP Has In Vitro Cytotoxicity against Lung Cancer at Low IC_50_ Values

In vitro cellular cytotoxicity of FWGP against lung cancer cell lines was determined using an MTS viability assay. IC_50_ values for different lung cancer cell lines were between 12 and 71 μg/mL and are presented in Table 1. The IC_50_ values for NSCLC cell lines include 71.74 μg/mL (A549), 20.99 ug/mL (Calu-1), and 12.88 μg/mL (H1975). Notably, against A549 cells, we found our protein formulation to be ~3 times more potent than FWGE, as measured previously by another group [5]. Although the FWGP IC_50_ for A549 was the highest among the cell lines we tested, we chose to focus the remainder of this study on A549 because: (a) it is among the most commonly used lines in NSCLC studies; and (b) we reasoned that in vivo efficacy vs. A549 would suggest that other cell types from Table 1 could potentially respond to FWGP treatment.

### 2.2. FWGP Induces Cell Cycle Arrest in A549 Cells

A549 cells treated with a low dose of FWGP (20 μg/mL) induced a greater proportion of cells into the G1/G0 phase when compared to the untreated control after 48 h (Table 2, Supplementary Figure S1). Cells treated with FWGP after 48 h presented with 57% in the G1 phase vs. 33% for untreated controls, indicating cell cycle arrest in the G1 phase. Reduction of cells in the S phase may indicate a slowing of DNA synthesis for cellular division.

### 2.3. FWGP Has Tumoricidal Activity with Minimal Toxicity as a Single Agent in Lung Cancer Xenografts

**Figure 1:**
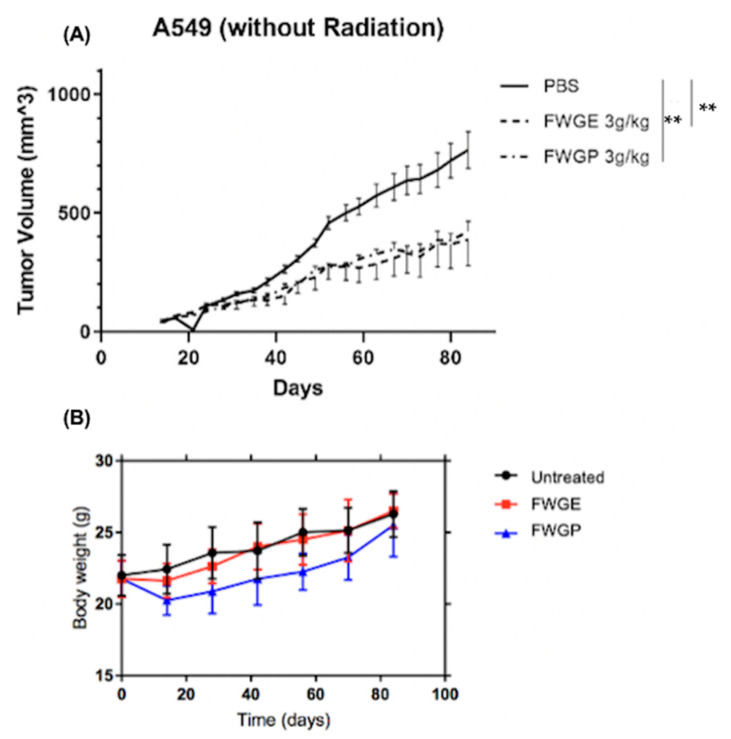
In vivo efficacy of FWGE and FWGP without pre-treatment irradiation. (**A**) A549 cells were implanted in nu/nu mice on both flanks of each mouse without radiation. Mice (n = 8 animals/group) were treated with either Avemar (FWGE) 3 g/kg gavage or FGWP 1.4 g/kg gavage 5 days/week for 8 weeks. (**B**) Body weight was measured to monitor possible toxicity throughout the study. Data represent the average ± standard error of the mean (SEM) of tumor volumes, (n = 16 tumors/group) as analyzed via ANOVA with Holm–Sidak multiple comparison. Statistical significance is indicated as ** *p* < 0.01.

The tumoricidal activity of FWGP was evaluated in vivo by treating nude mice with established bilateral flank A549 tumors. A549 cells were selected for their aggressive nature in vitro, and we expected any efficacy data against this model to be more impressive than selecting a more sensitive tumor type. Treatment began 2 days after tumor implantation and included FWGE (3 g/kg), FWGP (1.4 g/kg) or PBS control, daily by gastric lavage, 5 days/week for 8 weeks. Doses were selected based upon prior studies testing the combined efficacy of FWGE and various neoplastic agents in a Lewis lung murine model [16]. The FWGP dose was selected because it reflects the protein content of FWGE after ethanol precipitation. Tumor sizes were averaged by group and are shown in Figure 1. Although both FWGE and FWGP significantly inhibited tumor growth compared to the untreated control (Figure 1A), there was no significant difference between FWGE and FWGP (*p* = 0.4346). At the end of the study, the average tumor volumes were 763 ± 290 mm^3^ for the PBS group, 387 ± 321 mm^3^ for the FWGE group, and 422 + 159 mm^3^ for the FWGP group. The FWGE and FWGP treatment groups were both significantly different from the PBS group (both with *p* < 0.001).

**Figure 2:**
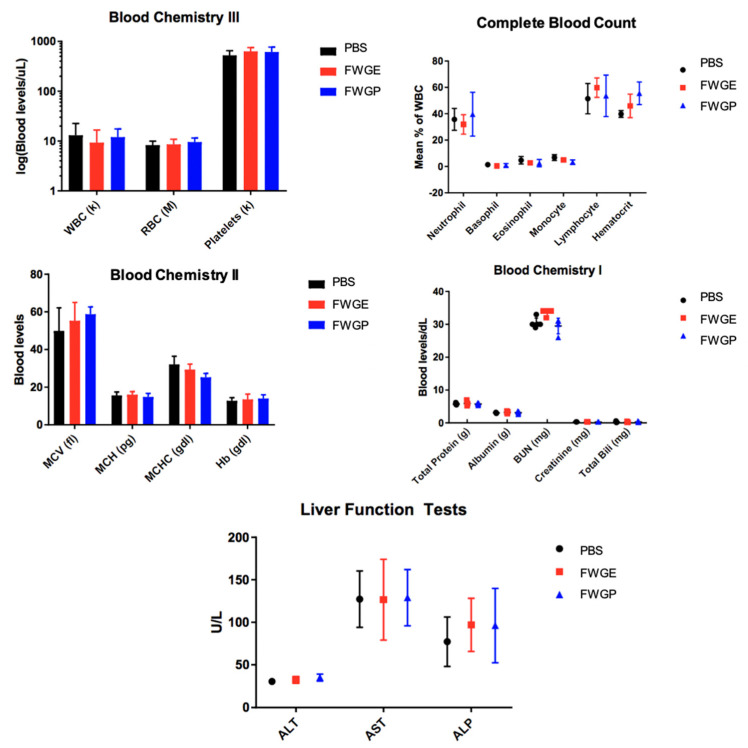
Blood samples were taken from 5 mice per treatment group at the end of the 84-day study and analyzed by the UC Davis Department of Comparative Pathology at the School of Veterinary Medicine. Error bars represent the standard deviation from the mean.

Body weight and blood chemistries were monitored to detect potential toxicity or side effects of treatment. All experimental groups demonstrated an increase in weight throughout the trial (Figure 1B). Additionally, there was no significant difference in blood levels of white blood cells (WBCs), red blood cells (RBC), or platelets. Leukocyte percentages were also consistent between experimental groups (Figure 2). A significant increase in hematocrit was observed in the FWGP group as compared to control group (56% vs. 39%, Figure 2). Serum chemistries were assessed for hepatic or renal toxicities. There were no significant differences in serum chemistries between treatment groups and untreated controls, except for a moderate increase in blood urea nitrogen (BUN) in the WGE group compared to both the untreated and FWGP groups (*p* < 0.0001), which may suggest mild dehydration (Figure 2). In total, the weight and blood chemistry data suggested that the treatments themselves were non-toxic.

In the second in vivo study, nude mice xenografts were again used to assess efficacy, but with the addition of pre-implantation radiation. Animals were treated as described above in the non-radiation study: separated into FWGE (3 g/kg), FWGP (1.4 g/kg), or PBS control groups and dosed 5 days per week. There was significant difference using 2-way ANOVA with a Holm–Sidak test between all treatment groups, FWGE, FWGP, and PBS (*p* value < 0.0001 for treatment vs. PBS and *p* value = 0.0035 for FWGP vs. FWGE). At the end of the study, the average tumor volume for the FWGP treated group was the smallest (470 + 175 mm^3^), followed by FWGE (583 + 256 mm^3^) and PBS (926 + 266 mm^3^). Unlike the first in vivo study, there was a significant difference between the FWGP and FWGE treatment groups.

## 3. Discussion

Despite substantial improvements in the efficacy of lung cancer treatments, most patients will suffer substantial treatment toxicity, and those with advanced disease will eventually succumb. Given the limited survival expectancy when diagnosed at advanced stages (70% of cases), and the taxing side effects of current standard therapies, there is a clear need for non-toxic and efficacious therapies for NSCLC.

Increasingly, patients are seeking alternative or complementary medicine either to treat their disease or prevent it [24]. One nutraceutical, FWGE, has demonstrated potential as a cytotoxic agent against multiple malignant cell lines in vitro such as colorectal carcinoma, melanoma, breast cancer, and lung cancer and has demonstrated in vivo tumor control in colon and melanoma models [4,5,10]. However, rigorous scientific analysis to confirm its efficacy in NSCLC is lacking. In this study, we demonstrate the anti-tumor activity of a unique protein fraction of FWGE, called FWGP, which exhibits direct cytotoxicity against NSCLC in vitro and in vivo as a single agent. Work from Imir et al. suggests one potential anti-tumor mechanism of FWGE against an NSCLC cell line may be impaired angiogenesis through decreased VEGF production, but data are limited [25]. Prior work has demonstrated multi-modal anti-tumor activity of FWGE with some conserved mechanisms in FWGP in both in vivo and in vitro lymphoma models [11,12,26]. In lymphoma cell lines, FWGP induced expression of cell cycle inhibitors—e.g., p53—resulting in cell cycle arrest, upregulated pro-apoptotic pathways (increased BAD and BAK expression, along with caspase 3/7 cleavage), and downregulated anti-apoptotic marker AKT [11,12]. These mechanisms are also seen in various tumor models treated with FWGE, suggesting that some of the activity is conserved. 

**Figure 3:**
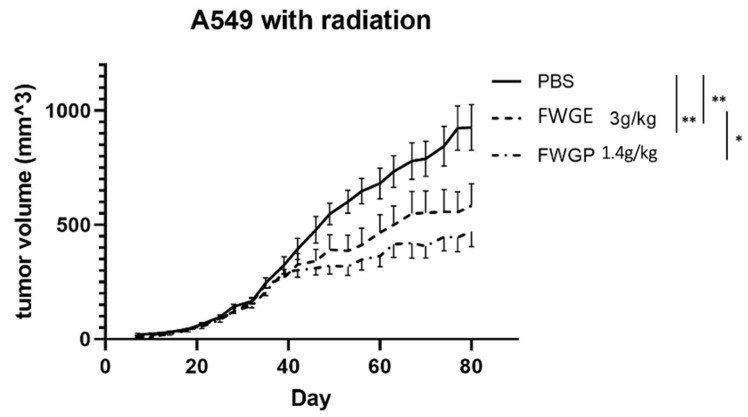
In vivo model of FWGE and FWGP with pre-implantation radiation. A549 tumors were established in nu/nu mice after receiving radiation (400 rad) on both flanks of each mouse, providing a total of 14 tumors for each experimental group. Data represent the averaged tumor volumes ± SEM for each mouse (n = 7 animals/group, ANOVA with Holm–Sidak multiple comparison). Statistical significance is indicated as * *p* < 0.05, ** *p* < 0.01.

In contrast to the first in vivo study, the significantly superior tumor control by FWGP compared to FWGE in our second mouse study (Figure 3) was intriguing. In the first study (Figure 1A), where the two treatments gave essentially identical results, the mice were not irradiated prior to tumor implantation. We hypothesize that the differential results in study 2 may be due to the recovery of the murine native immune cells, combined with their stimulation by FWGP. Prior studies suggest that despite radiation for murine tumor implantation, athymic nude mice begin to regain significant NK cell ex vivo cytotoxic activity (up to 50% of pre-radiation) between 2 and 7 weeks post radiation, whereas residual T cells may only regain 10–15% of their pre-radiation levels [27]. The FWGP-treated group in the irradiated mice began to show greater tumor control compared to FWGE approximately 40 days following irradiation, which could suggest that this effect was promoted by drug-mediated stimulation of the reconstituted NK cell compartment in the mice. This is consistent with our previous data suggesting that FWGP-mediated in vivo anti-lymphoma activity is likely a result of FWGP-mediated NK cell activation [12].

Presently, our data suggest that treatment with FWGE and FWGP inhibits tumor growth in NSCLC murine models with or without pre-implantation radiation, suggesting the active component within FWGE is retained in the protein formulation, FWGP. Further, the protein component retains or exceeds the potency of the crude FWGE extract demonstrated by in vivo efficacy at a lower dose—1.4 g/kg FWGP vs. 3 g/kg FWGE. Importantly, mice were able to receive daily doses of treatment without experiencing any overt treatment-related toxicities (Figure 1B and Figure 2). In human clinical studies, continuous treatment of FWGE conferred no additional treatment-related toxicity when added to adjuvant 5-FU regimens in colorectal cancer patients or in combination with dacarbazine in melanoma patients [14,28]. In a review of clinical data of FWGE treatment, patients across multiple cancer types receiving 8.5 g of FWGE daily for an average of 8–32 months experienced no treatment-related adverse events besides a few cases of diarrhea [29]. In fact, some patients who received FWGE in addition to chemotherapy agents reported reduced rates of chemotoxicity when compared to patients receiving chemotherapy alone. We believe that FWGP, being derived from FWGE, would have a similar non-toxic profile. Additionally, combination studies of FWGE with toxic chemotherapeutic agents such as docetaxel and cisplatin yield similar levels of ovarian or hepatocellular carcinoma cell killing while allowing for up to 10-fold lower doses of the chemotherapy [6,7]. Future studies will investigate the potential of FWGP combination regimens to provide similar efficacy while using lower doses of toxic chemotherapeutics. Given the poor outcome in patients with advanced stage NSCLC and the toxicity of currently available therapies, new therapeutics that are less toxic are needed. FWGP could be a promising natural agent that has minimal toxicity and demonstrated efficacy, and thus deserves further study in other preclinical models of lung cancer and clinical human trials.

Limitations: The goal of this initial study was to quantify tumor growth inhibition in two mouse models. Although the early data are encouraging, we acknowledge the limitations around interpreting anti-tumor mechanisms from athymic mice treated with FWGP. Evidence of immune activation was not performed in vivo, which would have supported our hypothesis as to why FWGP performed better in radiated vs. non-radiated mouse models and may have supported an additional therapeutic mechanism for FWGE and FWGP. Although our data suggest that FWGP induces cell cycle arrest in A549 cells (as is seen in cell lines from other studies), we do not have data to suggest which cellular proteins or pathways initiate this halting. Future studies will address these topics.

## 4. Materials and Methods

### 4.1. Production of FWGP and FWGE (Avemar)

Avemar was purchased from the manufacturer, Biropharma Ltd. (Budapest, Hungry) for crude FWGE and FWGP preparations. FWGE was prepared by reconstituting Avemar in phosphate buffered saline (PBS), pH 7.4, to a stock concentration of 300 mg/mL. FWGP was prepared by ethanol precipitation of Avemar. Briefly, Avemar was reconstituted in cold water at a ratio of 1 g Avemar to 10 g of water and stirred for 2 h at 4 °C. Supernatant was collected after centrifugation (9500× *g*, 4 °C, 15 min) and cold ethanol was added while stirring to a final concentration of 70% ethanol. The solution was maintained overnight at 4° before being centrifuged to collect the pellet (9500× *g*, 4 °C, 15 min). The pellet was reconstituted in PBS, pH 7.4, to a stock concentration of 140 mg/mL. Both reconstituted FWGE and FWGP were stored at 4 °C for immediate use or at −20 °C for long-term storage.

### 4.2. Cell lines and Primary Specimens

Lung cancer cell lines H1650 (CRL-5883), A549 (CCL-185), Calu-1 (HTB-54), H1975 (CRL-5908), HCC827 (CRL-2868) and H460 (HTB-177) were purchased from ATCC (Rockville, MD, USA) and grown in either RPMI-1640 (Gibco, 11875093, Nebraska, NY, USA) or DMEM (Gibco, 11965092) supplemented as recommended by ATCC with 10% heat-inactivated fetal bovine serum (Gibco A3840001) and 100 units/mL penicillin/100 g/mL streptomycin sulfate (Gibco 15140122) at 37 °C, 5% CO_2_ and 90% humidity. Fresh vials of cells were periodically thawed and utilized for in vitro experiments over time. For xenograft studies, cell lines were thawed and cultured for 7–10 days prior to tumor cell establishment. 

### 4.3. Cytotoxicity of FWGP against Lung Cancer Cell Lines In Vitro

Direct cytotoxic activity of FWGP was measured by incubating 5 × 10^4^ cells/well on 96-well plates in 100 μL culture medium with the indicated concentrations of FWGP for 72 h at 37 °C, with 5% CO_2_. Cell viability was assessed using an MTS-based assay (Promega, G3582) according to the manufacturer’s instructions and compared to untreated controls. Three replicate wells per condition were used in three independent experiments for each cell line tested. To derive IC_50_ values for each cell line treated with FWGP, treatment concentrations of FWGP were plotted against % cell viability to create a dose–response curve. To find the concentration of FWGP at which viability was 50% (IC_50_ values), these dose response data were fit to a linear regression model using GraphPad Prism software 9.1. 

### 4.4. Cell Cycle Arrest Assay with FWGP

Cell cycle analysis was performed with A549 cells treated with FWGP to determine the cytostatic effect of FWGP treatment as described previously [30]. Briefly, 5 × 10^4^ A549 cells were plated into a 12-well plate and allowed to adhere overnight. Media was removed and cells were incubated with or without 20 μg/mL FWGP in culture media. After 24 h and 48 h, cells were trypsanized, washed with PBS and fixed in 70% ethanol at −20 °C for at least 24 h. Cells were then washed with PBS to remove ethanol, stained with propidium iodide 1 μg/mL (Invitrogen, P1304MP) and RNAse A 20 μg/mL (Thermo Scientific, EN0531) for 30 min at room temperature before being acquired by flow cytometry. Samples were analyzed using a Becton-Dickinson FACSCalibur cytometer. Cell cycle analysis was performed using FlowJo software version 10.8.1 (Ashland, OR) using the Watson pragmatic model to determine cell cycle percentages.

### 4.5. Animals and In Vivo Studies

For xenograft experiments, female 6–8-week-old nu/nu mice (Harlan, Indianapolis, IN, USA) were kept in micro-isolation cages under pathogen-free conditions at the UC Davis animal facility in accordance with approved protocols under national and institutional guidelines for animal care and maintenance. For protocols including whole body irradiation (400 rad), A549 lung cancer cells (7 × 10^6^ in 100 μL PBS) were implanted subcutaneously on both the left and right flanks 3 days after irradiation. Treatment began 2 days after tumor inoculation and mice were subsequently divided into treatment groups (n = 7). For protocols without irradiation, mice were injected on both flanks with A549 cells (8 × 10^6^ in 100 μL pbs). Treatment began 2 days after implementation and mice were randomly divided into treatment groups (n = 8). Treatment (FWGE, FWGP or PBS) was administered by gavage 5 days/week for the entirety of the studies. Tumors were measured twice weekly using a digital caliper. Tumor volumes were calculated utilizing the formula: (length × width × depth) × 0.52. Mice were euthanized according to the method approved by the Institutional Animal Care and Use Committee (IACUC) if a tumor reached 15 mm in any dimension, if animals demonstrated signs of distress, and at the at the end of the 84-day study. Body weight was measured twice weekly for the first 28 days, then weekly for the rest of the 84-day study. Toxicity was evaluated from blood samples obtained post-mortem from 5 mice per treatment group and sent to the University of California Davis Comparative Pathology Laboratory for blood toxicity profiling. 

### 4.6. Statistical Analyses

In vitro cytotoxicity data were analyzed via a two-tailed, unpaired Student’s *t*-test. For experiments including 3 or more groups, data were analyzed using 2-way ANOVA with a Holm–Sidak test for multiple comparisons, as indicated in each figure. GraphPad Prism software 9.1 (San Diego, CA, USA) was used for all statistical analysis and representation. Statistical significance is indicated as * *p* < 0.05, ** *p* < 0.01, *** *p* < 0.001 and **** *p* < 0.0001. 

### 4.7. Ethics

All work with animals was performed in accordance with the national and international guidelines under protocols approved by the University of California Davis Institutional Animal Care and Use Committee (AAALAC accreditation #000029; PHS Animal Assurance #A3433-01; USDA Registration #93-R-0433).

## Figures and Tables

**Table 1:** Human cell lines treated with FWGP. Cells were incubated with various concentrations of FWGP to determine IC_50_ levels. IC_50_ doses were determined using GraphPad Prism software version 9.1 non-linear fit regression analysis.

Cell Line	Tumor Type	IC_50_ (μg/mL) (SD)
A549	Non-small cell lung cancer (NSCLC)	71.74 (±13)
H1650	Bronchoalveolar carcinoma	22.52 (±5)
Calu-1	NSCLC	20.99 (±8)
H1975	NSCLC	12.88 (±3)
HCC827	Lung adenocarcinoma	17.32 (±6)
H460	Large cell lung cancer	62.82 (±29)

**Table 2:** Cell cycle arrest for A549 cells treated with FWGP (20 μg/mL). 5 × 10^4^ A549 cells were plated overnight before being treated with FWGP or PBS in cell culture media for the time points indicated. Cells were harvested, stained with propidium iodide, and acquired by flow cytometry. Cell cycle analysis was performed using FlowJo software v10.8.1.

Time Point/Treatment	%G1	%S	%G2
Untreated	33.1	46.6	11.3
FWGP (20 μg/mL), 24 h	33.6	48.1	10.3
FWGP (20 μg/mL), 48 h	57.4	29.6	12.1

## Data Availability

Data are contained within the article.

## Supplementary Materials

The following supporting information can be downloaded at: https://www.mdpi.com/article/10.3390/cimb45090448/s1, Figure S1: Cell cycle analysis of A549 cells treated with FWGP.
