# Supplementary figures and images for: A Fermented Wheat Germ Extract Contains Protein Components Active against NSCLC Xenografts In Vivo
Source: Curr Issues Mol Biol. 2023 Aug 25;45(9):7087–96. doi: 10.3390/cimb45090448 (PMC10530145; doi:10.3390/cimb45090448)

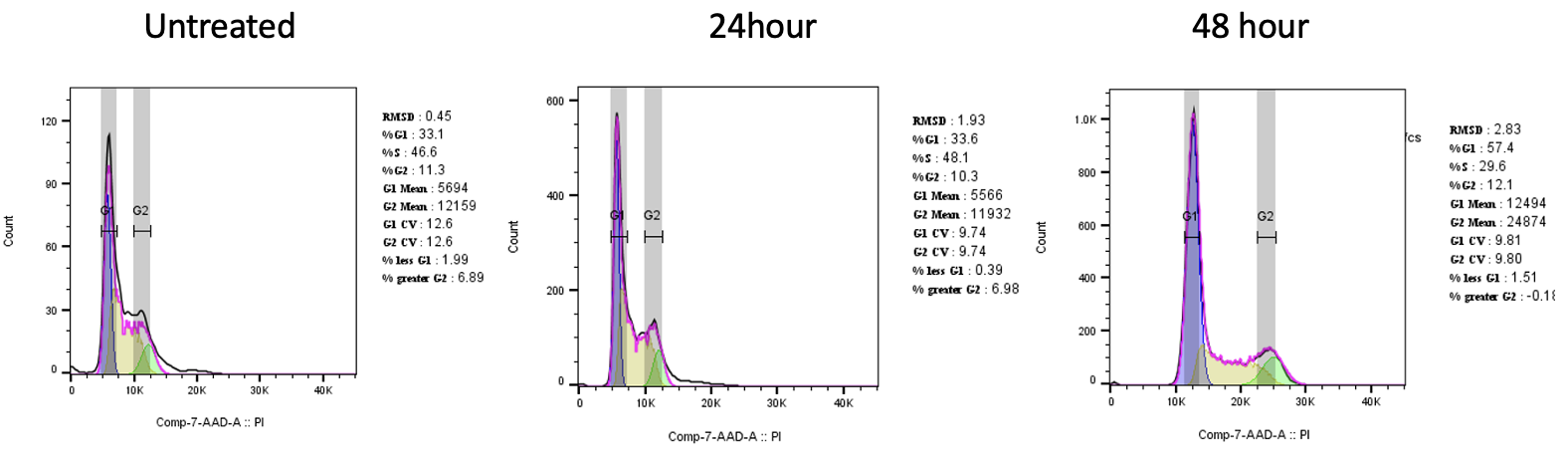

Supplement: Supplementary file 1 [file cimb-45-00448-s001.zip › cimb-2566631-supplementary.tiff]
